# Personal Experience with Fracture of the Hip1Read at the thirty-second annual meeting of the Medical Association of Central New York, at Syracuse, October 17, 1899.

**Published:** 1900-06

**Authors:** George Walton Greene

**Affiliations:** Auburn, N. Y.


### PERSONAL EXPERIENCE WITH FRACTURE OF THE HIP.[1]

By GEORGE WALTON GREENE, M. D., Auburn, N. Y.

ON THE third of July, 1897, I was so unfortunate as to fall from a ladder, striking with great force on the great trochanter of the right hip. Gentle hands quickly bore me to the house and laid me on the bed. Three surgeons were quickly summoned and proceeded to examine me. There was something peculiar, indescribable about the pain, something that I had once before experienced, which told me in unmistakable language that my hip was broken, and I told them so before they picked me up. After getting me under the influence of ether, they went carefully through all the various motions and measurements that are described in the books to be followed in injuries of the hip without finding anything. There was no apparent shortening; there was no eversion; there was no crepitation. A line drawn from the tuberosity of the ischium to the anterior superior spine of the ilium passed directly over the great trochanter of the femur. Nothing in fact to denote fracture.

Who could blame the physicians, all experienced men, for saying fracture did not exist? But remembering my being so positive in the matter, they very wisely put on a splint and extension. When visiting me the next day the occurrence of severe pain and the peculiar symptom known as sub sultustendonon, quickly settled the matter in their minds—a fracture did exist. Nor can I blame them for failing to find the fracture. I once had an exactly similar case. A man fell from a tree. A glance told me I had a fracture of the forearm; but his hip was different, the man suffered from pain, and insisted that "something was busted." But yet I was unable to detect it, and decided that fracture did not exist. I did not act so wisely as the physicians who attended my accident. I did not dress it as a fracture. However, when I visited him in the morning, I found his

1. Read at the thirty-second annual meeting of the Medical Association of Central New York, at Syracuse, October 17, 1899.

leg lying on the external maleolus, fully an inch shortening, and on the slightest motion marked crepitation. Then I began to realise that there was certainly "something busted."

These two cases prove to my mind conclusively one thing not mentioned in the books at least, so far as I am able to learn, viz.: that the opinion of the injured man must be given some weight, especially if he be a man of fairly good intelligence. In regard to the pain, I think this subject has been sadly neglected by authors. Stimson says in his valuable book while treating of symptoms, "Pain is sometimes so severe as to require a full dose of opium to relieve it," while under treatment he entirely ignores the subject. Hamilton says that the pain is sometimes so severe as to require opium by the mouth or the use of the hypodermic. The American Text-book of Surgery says practically the same thing.

I think that all present, who have treated a large number of cases of hip fracture, have witnessed the suffering, have heard the cries of pain, will agree with me that the subject deserves something more than a passing remark, especially if they have passed through the same ordeal themselves.

There are three distinct varieties of pain: First, the subsultus or contracture of the muscles crossing the fracture. The patient, worn out by suffering, begins to be calm, perhaps to doze. All at once there is a violent contraction of the muscles, the patient awakes with a scream. The slightest touch of the foot or bed-clothes, a cough or sneeze or even a long breath, will cause a renewal of these symptoms. The treatment of this condition is by extension and anodynes. Extension is put on to prevent shortening and relieve pain by tiring out the muscles, thus preventing tonic contraction. In my case I recovered with one and one-half inch shortening, the same amount as my other leg, without extension. In regard to pain in my case, it was an utter failure. If it ever becomes my duty to treat a similar case, I shall not hesitate to put on fifty or even sixty pounds, keeping the patient from slipping to the foot of the bed by a well-padded strap from beneath the shoulders to the head of the bed. Two or three days is generally sufficient to produce complete relaxation. But two or three days of suffering, such as accompanies such injuries, is a long time to wait. Is it any wonder that physicians resort to opium? But authorities do not say anything about the method of administration or the dose. It was a principle that I was taught when in college, that when you give medicine to produce a symptom, that unless you produce that symptom you fail. Pain being the symptom that you wish to overcome, in this instance you should give morphine, regardless of dose, until you allay the pain. In my own case one-eighth grain produced no result, one quarter of a grain only a temporary quiet; but one-half grain given by hypodermic produced a restful night. Think of it! Half a grain by hypodermic needle. That is equal to a grain taken by the stomach. There is one objection to the use of morphine or opium: it seriously constipates the bowels. The same condition affects the bladder and renders the use of the catheter necessary. So inactive were my bowels and the inability to use the necessary muscles that it was impossible for me to empty my bowels, even of flatus. Under such conditions you can see the serious objection to the use of opium. There is, fortunately, one salt of opium which, while retaining the narcotising quality of opium, does not produce this serious condition. I refer to codein, a drug that I think should be more largely used in general practice. If I am ever called to treat a fracture of the hip, I shall give it in doses anywhere from one-half to two grains, not being alarmed at the dose till the desired effect is reached—cessation of pain.

The second variety of pain is backache, which is entirely due to the confined position. This is the most unfortunate form to treat. No physician would be so unwise as to give opium, no matter how much the patient complained. I think this symptom is best treated by slight changes in the position. Put a pillow under the shoulder; in a short time remove it and put under the hips; when the patient begins to complain, withdraw it, and put under the small of the back. Put a hot water bag under one hip, and then under the other, and then under the small of the back All this will require the constant attention of an experienced nurse. But constant attention is the only way that the unfortunate victim can be made any way comfortable.

The third form is also due in general to the confined position. It is a sort of a neuralgic pain and differs materially from the agonising spasmodic jerking of the muscles. Sometimes in the ankle or the hip, more frequently in the knee; it comes on more frequently in the night, when the patient is worn out and exhausted, and sleep would be refreshing. A pillow or book should be placed under the knee or ankle or both, and thereby change the position. Many times during the long months of my suffering, I would say to my faithful wife, "Raise my knee by putting the book under it." After examining it she would say, "The book is already there"; then I would say "raise it higher by putting a small pillow on the book or take it out entirely." I had learned by experience that all I wanted was a change of position.

There is one point about fractures in general, where I think practitioners are seriously at fault. Not that authors on surgery are not posted on the question, but they do not lay stress enough on the fact. I refer to tight bandages. I know one young man who lost part of his hand, and another who was seriously threatened with gangrene of the ankle from this cause. In treating any fracture, I always lay particular stress on the fact that if the bandage is too tight they shall cut it. The first night after my accident, owing to swelling of my limb, I suffered severe pain in my ankle. I pleaded and beseeched them to cut the bandage, but to no avail. The doctor said nothing about cutting it, and if he thought it ought to be cut, he would have told them so. At last, at my earnest solicitation, they called Dr. Hyatt, who fortunately lived next door, and he immediately cut the adhesive plaster about the ankle. Relief was instantaneous.

After laying on my back for three months, we removed the extension and splints, under the belief that firm union had taken place. What was my surprise and disappointment, however, on a slight motion to feel crepitation. Notwithstanding the argument of my medical friends that it was all imagination, I would not be convinced. When well marked shortening occurred, they too gave in. Union had not taken place. We now called Dr. Creveling in consultation with Dr. Cheesman, and he proposed a plan that was new to me, which I think was entirely original with him. A short time afterward, I read an article by Dr. Senn, of Chicago, and Dr. Schaffer, of New York. Dr. Senn advocated putting a tourniquet around the hip with sufficient force to compress the fracture and get up enough irritation to cause union. Dr. Schaffer elevated the knee on an incline, putting a frame around the hip with a screw, by means of which firm pressure is produced on the great trochanter, but Dr. Creveling's method was the simplest. He advised, after padding the hips, to put a strong elastic bandage around the body and get up the same irritation as the others advised. Dr. Stimson, in the last Annual of Medicine, advised exactly the same method. Now I wish to say a few words about bony union, nonunion, and fibrous union. If, after three months in bed, the extension is removed and no immediate shortening takes place, if, when the patient gets up on crutches, the limb is painful and weak, but no crepitation is felt, then I think you could safely say that bony union was taking place; but if, on removing the extension, immediate softening takes place, possibly eversion, and on the slightest touch, well marked crepitation, you could with equal certainty say there was nonunion; but if, on removing the extension, no immediate shortening or eversion occurred, if when the patient got on crutches, there was crepitation which gradually diminished and finally ceased, if eventually the patient had a useful limb, then you would say that firm fibrous union had taken place. Such is my case.

DISCUSSION.

Dr. A. CLIFFORD MERCER, Syracuse, N.Y.—Just one point. The *x*-rays were not used in this fracture and it would seem from the experience which is being gathered, I think, in more places than we are aware of, that the *x*-ray is going to be the thing which will be helpful at one point in this experience where help was lacking for a while. In some of the hospitals, an enormous amount of work and experience is being gathered. Last November, in one of the Boston hospitals, I found they had made over 5,000 observations, and that is only a little of what is going on quietly, I imagine, all over the country everywhere.

But to the point. It happens that today, in one of the shop windows in Syracuse, are exhibited five radiographs. They are exhibited in a window in the University Building, the Thompson Art Store. Those five radiographs were made by Prof. Goodspeed, of the University of Pennsylvania, about the time of this fracture, I think. If it had only been in Philadelphia, there might have been some light thrown on it. Those radiographs were seen by Lord Kelvin while he was here and they are pronounced to be the finest that have been produced in the world; and if there are any members present who are interested in *x*-ray work, there is an opportunity to see the high water mark of *x*-ray work. I think, if you see these radiographs, you will say that if pictures of fractures can be made—these are normal pictures—as well as these normal radiographs show up, there will be no question about the diagnosis of fractures in future. They are so good that I wanted this opportunity to make the announcement so that you may see them, if you choose to.

